# Treating severe allergic asthma with anti-IgE monoclonal antibody (omalizumab): a review

**DOI:** 10.1186/2049-6958-9-23

**Published:** 2014-04-15

**Authors:** Gennaro D’Amato, Anna Stanziola, Alessandro Sanduzzi, Gennaro Liccardi, Antonello Salzillo, Carolina Vitale, Antonio Molino, Alessandro Vatrella, Maria D’Amato

**Affiliations:** 1 Division of Respiratory and Allergic Diseases, Department of Respiratory Diseases, High Speciality Hospital A. Cardarelli, Naples, Italy; 2 Committee of Ministry of Health on "Pollution, Climate and Respiratory Health, Naples, Italy; 3 Chairman Committee and Task Force on “Air pollution, climate change and allergic respiratory diseases” World Allergy Organization, Naples, Italy; 4 Division of Pneumology, Department of Respiratory Diseases, High Speciality Hospital “V.Monaldi” Naples and University of Naples Federico II, Naples, Italy; 5 Division of Respiratory Diseases, University of Salerno, Salerno, Italy

**Keywords:** Allergic asthma, Allergic respiratory diseases, Anti-IgE therapy, Monoclonal anti-IgE antibody, Omalizumab, Therapy of asthma, Urticaria

## Abstract

Increased asthma severity is not only associated with enhanced recurrent hospitalization and mortality but also with higher social costs.

Several cases of asthma are atopic in nature, with the trigger for acute asthma attacks and chronic worsening of inflammation being allergens inducing an immune, IgE mediated response.

Anti-inflammatory treatments are effective for most of asthma patients, but there are subjects whose disease is incompletely controlled by inhaled or systemic corticosteroids and these patients account for about 50% of the healthcare costs of asthma.

Omalizumab is a biological engineered, humanized recombinant monoclonal anti-IgE antibody developed for the treatment of allergic diseases and with clear efficacy in adolescent and adult patients with severe allergic asthma. The anti-IgE antibody inhibits IgE functions blocking free serum IgE and inhibiting their binding to cellular receptors. By reducing serum IgE levels and IgE receptor expression on inflammatory cells in the context of allergic cascade, omalizumab has demonstrated to be a very useful treatment of atopic asthma, improving quality of life of patients with severe persistent allergic asthma that is inadequately controlled by currently available asthma medications. Several trials have demonstrated that this therapy is well tolerated and significantly improves symptoms and disease control, reducing asthma exacerbations and the need to use high dosage of inhaled corticosteroids.

## Introduction

A body of evidence suggests that bronchial asthma and other allergic diseases have become more common worldwide in recent years and are recognized as a higly prevalent health problem in the developed and developing world [1-6]. It is estimated that more than 50% of asthma has an allergic background and about 50% of patients with severe asthma have allergic-atopic asthma [2,6], although many previously published data demonstrated that the severe phenotype is less frequent in atopic adult-onset asthma [6-8].

## Review

It is well known that immunoglobulin E (IgE) antibodies, Th2 derived cytokines and eosinophils play a major role in the development of chronic airway inflammation, which is observed even in subjects with mild disease [9-12]. Airway inflammation plays a central role in the pathogenesis of bronchial asthma and is associated with an increase in airway responsiveness to several trigger factors such as aeroallergens which induce bronchoconstriction in atopic asthma patients, acting sometimes in cooperation with other trigger factors such as viruses and air pollution.

The development of inflammation in asthma involves a complex array of several inflammatory mediators that promote the recruitment and activation of various different immune cells and regulate inflammatory cell trafficking into the lungs.

Activation of chemokine receptors triggers multiple cascades of intracellular signaling events that lead to recruitment and activation of immune effector cells. The inhibition of specific chemokines and receptors could prevent the excessive recruitment of leukocytes to sites of inflammation.

Elevated serum levels of specific IgE towards common environmental allergens are a key component in the pathogenesis of allergic asthma. IgE antibodies cause chronic airway inflammation through effector cells such as mastcells, basophils etc, activated via high-affinity (FcϵRI) or low-affinity (FcϵRII) IgE receptors.

Current treatment for asthma suggested by Global Initiative for Asthma (GINA) guidelines includes several reliever and controller drugs, in particular corticosteroids which reduce recruitment and activation of inflammatory cells in the airways [13]. The available anti-asthma treatments are effective for most of these patients. However, there are asthmatic subjects who continue to experience severe debilitating disease, since their bronco-obstruction is incompletely controlled by inhaled or systemic corticosteroids associated with other drugs such as beta-2 agonist bronchodilators (short and long-acting), antileukotrienes etc. Moreover, increased asthma severity is not only associated with enhanced recurrent hospitalisation and mortality within one year of initial hospitalisation, but also with higher economical and social costs [14].

IgE antibodies have been viewed as a target for immunological drug development in asthma, and a number of strategies aimed at inhibiting its proinflammatory action have been developed, despite an increase in recent years in the availability of drugs used for asthma therapy.

### Mechanisms of action of omalizumab

Therapeutic anti-IgE antibodies, omalizumab, able to reduce free IgE levels avoiding the binding of IgE to Fcϵ RI and the following development of allergic reaction (crosslinking IgE and triggering degranulation and syntesis of new-generated chemical mediators of IgE-sensitized cells) are now widely used in the therapy of allergic bronchial asthma [15-34]. This anti-IgE monoclonal antibody (omalizumab) binds IgE at the same site of Fc fragment defined Cϵ3 domain as these antibodies bind Fcϵ RI and FcϵRII. Consequently, IgE effector functions are inhibited, because the IgE binding to high-affinity receptors on IgE effector cells is blocked, as well as the following activation of mast cells and basophils [15-35]. In other words, in allergic subjects omalizumab prevents the activation of cellular response and the occurrence of asthma symptoms. Omalizumab expresses a high degree of isotype specificity and can neutralize serum free IgE without affecting other antibody classes.

Studies in patients with atopic asthma showed that anti-IgE antibodies decrease serum IgE levels in a dose-dependent manner and allergen-induced bronchoconstriction during both the early and late-phase responses to inhaled allergen [18-20].

Serum free IgE are rapidly reduced after omalizumab administration and the expression of high-affinity receptors is significantly reduced after three months treatment [34]. Nevertheless, when omalizumab is withdrawn after few months of therapy, the serum IgE levels return to pretreatment values as well as the number of IgE receptors on the basophils surface. This structural “involution” reflects the trend of the symptoms related to the asthmatic disease, leading patients to increase the dosage of standard therapy [34-44].

Nopp *et al*. investigated the long-term efficacy of 6-year-therapy with omalizumab in 18 patients with severe IgE-mediated asthma 1 year [35] and 3 years [36] after the withdrawal of omalizumab. In both cases the Authors documented the stabilization of the asthma-related symptoms, similar to that observed during the treatment period with omalizumab, as well as the downregulation of basophil allergen sensitivity.

The treatment with omalizumab should be potentiated by specific immunotherapy, which is active by using other immunologic mechanisms [45].

Recently, two studies investigated also the potential effects of omalizumab on airway remodeling. Hoshino et al. [46] evaluated the effects of omalizumab on airway wall thickening using Computed Tomography (CT). Thirty patients with severe persistent asthma were randomized to conventional therapy with (n = 14) or without omalizumab (n = 16) for 16 weeks; the airway dimensions were assessed by a validated CT technique. This study showed that 16 weeks of treatment with omalizumab reduced airway wall thickness. The change in wall thickness was associated with a marked reduction in sputum eosinophils, and improvement in pulmonary function and quality of life [46].

Riccio et al. [47] investigated the effect of long–term anti**-**IgE on the thickening of the reticular basement membrane (RBM) and eosinophil infiltration in bronchial biopsies from 11 patients with severe persistent allergic asthma before and after (12 months) treatment with omalizumab. The study showed that a substantial proportion of severe asthmatics reduced the original bronchial RBM thickness and eosinophil infiltration after one-year treatment with anti-IgE [47].

### Omalizumab in allergic asthma

In several clinical controlled trials omalizumab resulted to be able to reduce asthma-related symptoms, to decrease corticosteroid use and to improve quality of life of asthmatic patients [28-44,48-51] and some of these studies show the benefits of anti-IgE as add-on therapy in patients with severe persistent asthma who are inadequately controlled by anti**-**asthma pharmacological therapy (Additional file 1).

The anti-IgE approach to asthma treatment has several advantages, including concomitant treatment of other IgE-mediated diseases and a favorable side-effect profile regardless of the type of allergic sensitisation [28-34,41-44,48]. Omalizumab was shown not only to inhibit mast cell and basophil responses but also to have inhibiting effect on the inflammatory cells, such as eosinophils, T lymphocytes and B lymphocytes which are fundamental to the chronic inflammatory response in allergic diseases such as asthma. This background places anti-IgE therapy firmly in the domain of an anti-inflammatory treatment for chronic allergic disease, with effect on multiple cell types.

### Omalizumab in severe asthma

Severe or refractory asthma remains a frustrating disease for both patients and the clinicians treating them. Severe asthma has been defined as persisting symptoms due to asthma despite high-dose inhaled steroids (1,000 mcg beclometasone dipropionate or equivalent) plus long-acting beta**-**2 agonists, with the requirement for either maintenance systemic steroids or at least two rescue courses of steroids over 12 months and despite trials of add-ons such as leukotriene-receptor antagonist or theophylline.

The Global Initiative for Asthma (GINA) [13] guidelines for patients with severe persistent asthma (step 5 therapy) recommend the use of high-dose inhaled corticosteroids plus a long-acting beta**-**2 agonist (LABA), and, if required, one more additional controller. Currently several studies showed benefits of omalizumab as add-on therapy in patients with severe persistent asthma who are inadequately controlled, despite best available therapy.

The INNOVATE (INvestigatioN of Omalizumab in seVere Asthma TrEatment) study was specifically designed to evaluate the efficacy and safety of add-on therapy with omalizumab in this difficult asthma population [43].

In the INNOVATE trial were enrolled patients aged 12-75 years with severe persistent allergic asthma (GINA step 3 or 4 clinical features despite step 4 therapy). The primary efficacy variable was the rate of clinically significant asthma exacerbations (defined as a worsening of asthma symptoms requiring treatment with systemic corticosteroids). A total of 419 patients were included in the efficacy analyses (omalizumab, n = 209; placebo, n = 210). The rate of clinically significant asthma exacerbations, after adjusting for an observed imbalance in asthma exacerbation history prior to randomization, was significantly reduced by 26.2% with omalizumab versus placebo (0.68 and 0.91, respectively; p = 0.042).

Compared with placebo, treatment with omalizumab significantly reduced the rate of severe asthma exacerbations (0.24 vs 0.48, p = 0.002) and the rate of total emergency visits for asthma (0.24 vs 0.43, p = 0.038). Significantly greater improvements were achieved with omalizumab compared with placebo in Asthma Quality of Life Questionnaire (AQLQ) scores (overall and individual domains), with a significantly greater proportion of patients receiving omalizumab achieving a clinically meaningful (> 0.5-point) improvement from baseline compared with placebo recipients (61% and 48%, respectively; p = 0.008).

Several “real life” studies confirmed the efficacy and tolerability of omalizumab in severe persistent allergic asthma patients.

One trial was an observational study performed in France [44]. The second one was a prospective post-marketing surveillance trial that evaluated the efficacy and tolerability of omalizumab in real-life in Germany [48], and the third study was a prospective multicenter real-life study conducted in Belgium, the PERSIST study [49], the fourth was a small questionnaire-based observational study in 65 patients in the UK, who had continued with omalizumab therapy beyond 16 weeks, conducted by Niven et al. [50].

In the first study, the authors evaluated 154 patients. The analysis performed during the treatment period and compared to the previous year, showed that patients with a follow up of at least 5 months experienced 62% fewer exacerbations requiring oral corticosteroids, 65% fewer emergency department visits and 29% fewer hospitalisations per year. Korn and co-workers [48] reported the results of the observation of 280 patients followed up for 6 months. After 6 months of specific anti-IgE therapy, omalizumab was demonstrated to reduce the daily (−76%) and nocturnal symptoms (−84%), exacerbations (−82%), unscheduled medical assistance (−81%), hospitalisations (−78%) and increase quality of life (Mini-AQLQ: score increase from 2.9 to 4.5). Overall, efficacy of omalizumab was rated as excellent or good by the majority of physicians (82%) and patients (86%).

In the PERSIST Study Brusselle and co-workers [49] evaluated the 15- and 52-week effectiveness of add-on omalizumab treatment in 158 enrolled subjects. After 16 weeks of therapy, a good/excellent global evaluation of treatment effectiveness (GETE) was achieved by > 82% (p < 0.001), the total AQLQ scores improved in > 82% by > 0.5 points (p < 0.001) and > 91% of the subjects were exacerbation-free. At 52 weeks, the same results were achieved by > 72% (p < 0.001), > 84% (p < 0.001) and > 65% (p < 0.001), respectively. In addition, a significant reduction in healthcare utilization compared the year prior to treatment was observed.

Niven et al. [50] found that out of 33 patients taking oral corticosteroid at baseline, 18 (54.5%) had reduced their oral corticosteroid and 8 (24.2%) had stopped oral corticosteroid altogether. The mean relative reduction in oral corticosteroid dose from baseline was 49% (22.6–11.6 mg, prednisolone equivalent).

All these studies showed that anti-IgE treatment has a reassuring safety profile. It is tolerated, and its overall adverse event profile is similar to that of placebo (Additional file 2).

### Safety and tolerability

In a review Corren and co-workers [51] evaluated the safety of omalizumab in a pooled analysis of data from 15 randomized multicentric studies involving more than 7,500 patients (adults, adolescents and children). All patients suffered from severe persistent allergic asthma and the majority of them received omalizumab for almost 24 weeks at the dose of 150-300 mg every four weeks or 225, 300 or 375 mg every two weeks. In all studies the number of adverse events (AEs) was similar between groups and the majority of AEs were mild or moderate. The most frequent AE observed in both groups was nasopharyngitis; no difference indicative of omalizumab specific tossicity was detected between groups. The only AE with > 2% difference between groups was sinusitis, observed in 10.1% of patients treated with omalizumab and in 12.2% in the placebo groups. The assessment of laboratory parameters did not show any significant effect of omalizumab on blood cells counts, renal and liver function; however, no data exist about patients with previous renal or hepatic impairment treated with omalizumab, thus caution should be used in administering the drug in these sets of patients. This review confirmed that add-on omalizumab is an effective and well tolerated treatment in patients with severe IgE-mediated asthma, and its cost-effectiveness is similar to other chronic disease biologics. Furthermore, the same authors highlight that, despite its cost, omalizumab used as an add-on therapy in this setting of patients improves quality-adjusted survival (QALYs) at an increase in direct medical costs, and that this value is directly related to the duration of the therapy.

Several other studies have been recently published. In the trial of Busse et al. [52] research has underscored the effects of exposure and sensitization to allergens on the severity of asthma in children and when added to a regimen of guidelines-based therapy for inner-city children, adolescents, and young adults, omalizumab further improved asthma control, nearly eliminated seasonal peaks in exacerbations, and reduced the need for other medications to control asthma.

In the study of Braunstahl et al. [53] by physician’s GETE, 69.9% of patients were responders to omalizumab after 16 weeks. The proportion of patients with no clinically significant exacerbations increased from 6.8% during the 12 month pre-treatment period to 54.1% and 67.3% at months 12 and 24, respectively. Symptoms and rescue medication use at month 24 were reduced by > 50% from baseline. Maintenance oral corticosteroids use was lower at month 24 (14.2%) compared with month 12 (16.1%) and baseline (28.6%). Overall, the results from eXpeRience registry indicate that omalizumab was associated with improvements in outcomes in patients with uncontrolled persistent allergic asthma and that these improvements were consistent with the results of clinical trials. However, omalizumab is effective in treating severe asthma also in patients with severe cardiovascular complications [54].

### Omalizumab in other respiratory and non-respiratory diseases

In addition to asthma, omalizumab has been investigated in treatment of various other diseases. However, there is suggestion for use of omalizumab also in intrinsic asthma [54-60].

The efficacy of omalizumab was also observed in patients with severe occupational asthma, regardless of whether the causative agent was high molecular weight (HMW) or low molecular weight (LMW) [61]. This is particularly relevant for patients who are unable to avoid allergen exposure in their place of work.

## Conclusions

Results of several large randomized trials have established omalizumab as an effective and well tolerated treatment for use as add-on therapy in patients with severe persistent allergic asthma. Several trials have demonstrated that this therapy is well tolerated and significantly improves symptoms and disease control, reducing asthma exacerbations and improving quality of life of asthma patients.

## Competing interests

The authors declare that they have no competing interests.

## Authors’ contributions

None

## Supplementary Material

Additional file 1: Allergic asthma subjects treated with omalizumab in the world and in Italy. Click here for file

Additional file 2: Clinical usefulness of omalizumab in allergic asthma patients. Click here for file
