# Supplementary material for: Treating severe allergic asthma with anti-IgE monoclonal antibody (omalizumab): a review
Source: Multidiscip Respir Med. 2014 Apr 15;9(1):23. doi: 10.1186/2049-6958-9-23 (PMC4113133; doi:10.1186/2049-6958-9-23)
Supplement: Additional file 1: — Allergic asthma subjects treated with omalizumab in the world and in Italy. [file 2049-6958-9-23-S1.pdf]

Additional file 1. Allergic asthma subjects treated with omalizumab in the world and in Italy

At present there are about 60,000 asthmatic patients treated with omalizumab in the world. In Italy the first two patients were treated contemporaneously in December of the year 1998 in Naples (High Speciality Hospital A.Cardarelli) and in Genova (Institute of Respiratory and Allergic diseases of the University). 2,400 asthmatic patients have been treated with omalizumab to date and currently patients treated are about 1900.
