# Supplementary material for: Treating severe allergic asthma with anti-IgE monoclonal antibody (omalizumab): a review
Source: Multidiscip Respir Med. 2014 Apr 15;9(1):23. doi: 10.1186/2049-6958-9-23 (PMC4113133; doi:10.1186/2049-6958-9-23)
Supplement: Additional file 2: — Clinical usefulness of omalizumab in allergic asthma patients. [file 2049-6958-9-23-S2.pdf]

Additional file 2. Clinical usefulness of omalizumab in allergic asthma patients

Omalizumab in clinical studies in allergic asthma patients showed to be able:

- To decrease IgE-induced bronchoconstriction during both the early and late- phase responses to inhaled allergen during the bronchial provocation tests.
- To reduce allergic asthma exacerbations regardless of the type of allergic sensitisation.
- To have a corticosteroid sparing effect.
- To reduce the use of bronchodilators.
- To improve the nasal symptoms in subjects with allergic rhinitis associated with asthma.
- To improve symptoms of urticaria and atopic dermatitis.
- To improve quality of life in patients with asthma, also in those with severe persistent allergic asthma that is inadequately controlled by currently available asthma medication.
